# Demultiplexer of Multi-Order Correlation Interference in Nitrogen Vacancy Center Diamond

**DOI:** 10.3390/ma14226745

**Published:** 2021-11-09

**Authors:** Xinghua Li, Faizan Raza, Yufeng Li, Jinnan Wang, Jinhao Wang, Hasnain Ali, Luyuan Wang, Yuan Zhao, Yanpeng Zhang

**Affiliations:** 1 Key Laboratory for Physical Electronics and Devices of the Ministry of Education and Shaanxi Key Lab of Information Photonic Technique, Xi’an Jiaotong University, Xi’an 710049, China; lixinghua1989@126.com (X.L.); faizanraza17@outlook.com (F.R.); 1543702761@stu.xjtu.edu.cn (Y.L.); hontkingwang@163.com (J.W.); 15399304754@sina.com (J.W.); hasnainali786@stu.xjtu.edu.cn (H.A.); wly0802@stu.xjtu.edu.cn (L.W.); 2 Key Laboratory of Time and Frequency Primary Standards, National Time Service Center, Chinese Academy of Sciences, Xi’an 710600, China

**Keywords:** nitrogen vacancy center diamond, optical demultiplexer, quantum computing and communications

## Abstract

We reported the second- and third-order temporal interference of two non-degenerate pseudo-thermal sources in a nitrogen-vacancy center (NV^−^). The relationship between the indistinguishability of source and path alternatives is analyzed at low temperature. In this article, we demonstrate the switching between three-mode bunching and frequency beating effect controlled by the time offset and the frequency difference to realize optical demultiplexer. Our experimental results suggest the advanced technique achieves channel spacing and speed of the demultiplexer of about 96% and 17 ns, respectively. The proposed demultiplexer model will have potential applications in quantum computing and communication.

## 1. Introduction

From Feynman’s point of view, interference is at the heart of quantum physics, and contains the only mystery of quantum physics [1]. Dirac argued that each photon inter-feres only with itself. Interference between different photons never occurs [2]. In the case of the generation of paired photons, a similar statement can be made for interference of two photons, in which superposition comes from the pair of photons jointly measured (analogous definition of Dirac) sharing the same energy level, such as in multi-wave mixing [3,4,5]. However, Paul considered Dirac’s statement to be limited to first-order coherence [6]. In [7,8], the second-order temporal and spatial coherence from two independent sources (coherent, pseudo thermal, and laser-photon, among others) is extensively studied using the “Hong-Ou-Mandel (HOM) dip” or “Shih-Alley dip” interference phenomenon [9,10,11]. Until now, no one has proposed the third-order temporal coherence from two independent sources of thermal fluorescence (FL). In this article, we have discussed the second- and third-order temporal interference between pseudo-thermal sources in the NV^−^ center in a diamond. The support for this idea comes from unified interpretation for the second-order subwave-length interference based on Feynman’s path-integral theory from both coherent and thermal sources [12,13,14] and their indistinguishability [14,15].

In the NV^−^ center, two adjacent sites in the diamond’s tetrahedral lattice of carbon atoms are altered. One site has an empty space instead of a carbon, and the other site has a nitrogen atom. Electrons orbit in the vacancy and around the adjacent four atoms and carry a spin that quantum applications can exploit. Doping more nitrogen atoms near the NV^−^ provides a system of coupled qubits that enables logic processing [16]. The NV^−^ center has important applications, such as in quantum registers. Researchers have demonstrated quantum registers in milestone room temperature built upon the NV^−^ electronic spin and proximal N and 13C nuclear spins [17,18].

In this paper, we investigate the interference in intensity–noise correlation by treating multi-order fluorescence generated from a negatively charged nitrogen-vacancy (NV^−^) center as a pseudo-thermal source. The resonant and non-resonant FL emissions serve as sources projected onto the beam splitter following Feynman’s path. At first, source indistinguishably is achieved under a dressed state picture of the diamond NV^−^ center, and later, path indistinguishability is introduced through time offset to study the interference phenomenon emerging from the NV^−^ center. The findings of these investigation are helpful to understand the emerging interference from the NV^−^ center at a low temperature. The results proposed for optical demultiplexer are based on the channel spacing and switching speed obtained from correlation and can be controlled by the time offset and frequency of incident beam.

## 2. Materials and Methods

**Figure 1:**
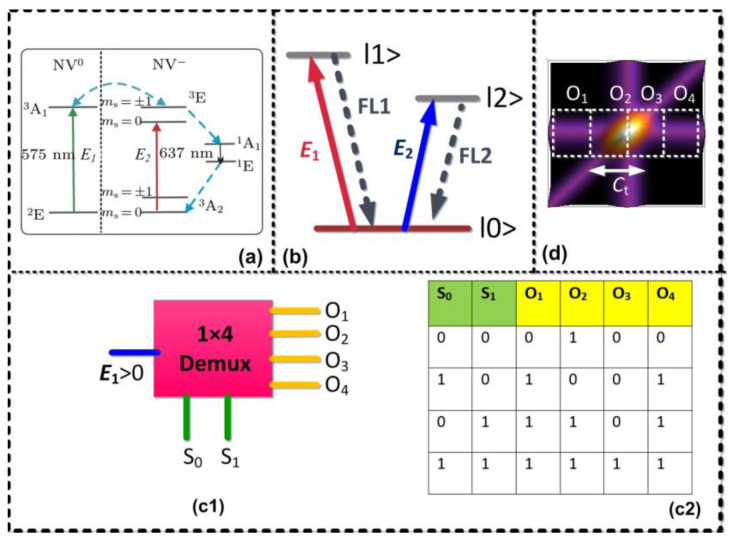
(**a**) Energy level diagram of NV^−^ interacting with laser pumping fields. (**b**) Three-level (V-type) atomic system in NV^−^ and laser coupling configuration. (**c1**) One to four demultiplexers with two selection lines (S_0_ and S_1_) to determine one of the four outputs (O_1_–O_4_). (**c2**) The corresponding truth table for the proposed demultiplexer. (**d**) Theoretical simulation of the third-order correlation function, where bright and dark state represents “Logic 1” and “Logic 0”, respectively. In this device, the positions on the correlation curve labeled as O_1_–O_4_ were defined as the four-output terminal and C_t_ is controllable channel bandwidth.

**Figure 2:**
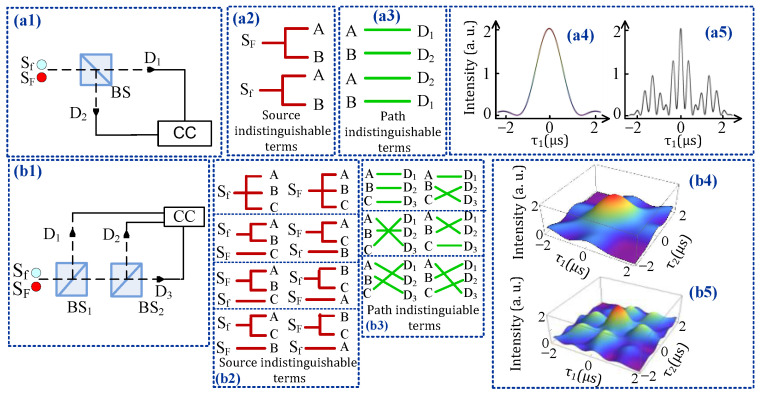
(**a1**,**b1**) Experimental setup to measure second- and third-order temporal intensity–noise correlation function of pseudo-thermal light, respectively. SF and Sf: pseudo-thermal sources. BS: 1:1 non-polarizing beam splitter. D: Detector. (**a2**,**b2**) Source indistinguishable terms for the second- and third-order correlation, respectively. (**a3**,**b3**) Possible path indistinguishable terms for second- and third-order correlation, respectively. (**a4**,**a5**) and (**b4**,**b5**) Calculated second- and third-order temporal correlation function, respectively.

The diamond NV^−^ center can be treated as a three-level electronic system having a ground triplet state ^3^A_2_, a triplet excited state ^3^E, and an intermediate singlet state ^1^A_1_. The two triplet states ^3^A_2_ and ^3^E are split into $|m_{s}=0\rangle$ and $|m_{s}=\pm 1\rangle$ fine-structure levels, as showed in Figure 1a. The energy difference between $|m_{s}=0\rangle$ and $|m_{s}=\pm 1\rangle$ for ^3^A_2_ is D = 2.8 GHz, while for the excited state, ^3^E is D = 1.42 GHz [19]. We considered a V-type three-level system |0〉→|1〉 from these fine-structure levels in Figure 1b. Figure 1(c1) shows the model of the demultiplexer with two selection lines (S_0_ and S_1_) to determine one of the four outputs (O_1_–O_4_) and Figure 1(c2) shows the truth table for the proposed demultiplexer.The sample used in our experiment is a <100> oriented crystal diamond, contains less than 5 ppb nitrogen concentration, and typically has less than 0.03 ppb NV^−^ concentration. The sample was held in cryostat; the cryostat temperature was maintained at 77 K by flowing liquid nitrogen. We used two tunable dye lasers (narrow scan with a 0.04 cm^−1^ linewidth) pumped by an injection-locked single-mode Nd/YAG laser (Continuum Powerlite DLS 9010, 10 Hz repetition rate, 5 ns pulse width, DLS represents dynamic light scattering), used to generate the pumping fields ***E*_1_** ($\omega_{1}$,$\Delta_{1}$) and ***E*_2_** ($\omega_{2}$,$\Delta_{2}$) with the frequency detuning $\Delta_{i}=\omega_{mn}-\omega_{i}$ (*i* = 1, 2), where $\omega_{mn}$ is the corresponding transition frequency between energy levels |m〉 and |n〉 and $\omega_{i}$ (*i* = 1,2) is the laser frequency. The input beams ***E*_1_** (575 nm) and ***E*_2_** (637 nm) are coupled to the transition |0〉→|1〉 and |0〉→|2〉, respectively, to generate the fourth-order fluorescence signals S_f_ and S_F_. The acquisition time of the experiment is 100 ms. In Figure 2(a1), both the S_f_ and S_F_ signals pass through a beam splitter (BS) and mutual interference occurs, after which they are detected by detectors D_1_ and D_2_. In Figure 2(b1), the fluorescence signals are divided into three subsequent parts through two beam splitters (BS_1_ and BS_2_), and then detected by three detectors D_1_, D_2_, and D_3_. 

## 3. Theoretical Model

By opening ***E*_1_** and ***E*_2_** fields, the fourth-order fluorescence signal generated via the perturbation chain $\rho_{00}^{\left(0\right)}\overset{E_{1}}{\to }\rho_{10}^{\left(1\right)}\overset{E_{1}^{*}}{\to }\rho_{00}^{\left(2\right)}\overset{E_{2}^{}}{\to }\rho_{20}^{\left(3\right)}\overset{E_{2}^{*}}{\to }\rho_{22}^{\left(4\right)}$ in a three-level V-type system (*E*_1_* and *E*_2_* are the conjugate fields of ***E*_1_** and ***E*_2_** fields, respectively.), the formula of density matrix element $\rho_{22}^{\left(3\right)}$ can be expressed as follows [20]:

(1) $$\rho_{22}^{\left(4\right)}=\frac{{\left|G_{1}\right|}^{2}{\left|G_{2}\right|}^{2}}{(\Gamma_{10}+i\Delta_{1}+\frac{|G_{2}{|}^{2}}{i\left(\Delta_{1}-\Delta_{2}\right)+\Gamma_{12}}+\frac{|G_{2}{|}^{2}}{\Gamma_{00}})(\Gamma_{20}+i\Delta_{2})(\Gamma_{00}+\frac{|G_{2}{|}^{2}}{i\Delta_{2}+\Gamma_{20}})\Gamma_{22}}$$

where $G_{i}=-\mu E_{i}/\hbar$ is the Rabi frequency of ***E_i_*** with the electric dipole matrix elements $\mu_{ij}$ of levels |*i*〉 and |*j*〉, and $\Gamma_{ij}$ is the transverse decay rate. The temporal intensity of FL emission is given as $I\left(t\right)=\rho_{FL}e^{-\Gamma_{FL}t}$. The lifetime of the measured *FL* signal includes the coherence process between two levels |*i*〉 and |*j*〉, which can be described as decoherence rate $\Gamma_{ij}=(\Gamma_{i}+\Gamma_{j})/2$(*i*, *j* = 0,1,2,3). Here, $\Gamma_{i/j}=\Gamma_{\mathrm{pop}}{+\Gamma }_{\mathrm{ion}-\mathrm{spin}}{+\Gamma }_{\mathrm{ion}-\mathrm{ion}}{+\Gamma }_{\mathrm{phonon}}{-\Gamma }_{\mathrm{dressing}}$, where $\Gamma_{\mathrm{pop}}={\left(2\pi T_{1}\right)}_{ij}^{-1}$ (the population decay time (T_1_)) depends on the location of the energy-level in phase-space, $\Gamma_{\mathrm{ion}-\mathrm{spin}}$ relates to the ion-spin coupling effect of the individual ion, $\Gamma_{\mathrm{ion}-\mathrm{ion}}$ is determined by the interaction among two charge states of the NV^−^ center, $\Gamma_{\mathrm{phonon}}$ is related to the temperature of the sample, and $\Gamma_{\mathrm{dressing}}$ is related to the dressing laser. The last four terms ($\Gamma_{\mathrm{ion}-\mathrm{spin}}$,$\Gamma_{\mathrm{ion}-\mathrm{ion}}$, $\Gamma_{\mathrm{phonon}}$, and $\Gamma_{\mathrm{dressing}}$) are components of the dephasing or coherence time, T_2_*. In detail, by taking the dressing term into account, one can obtain (2πT_1_)_11_^−1^ = (2πT_1_)_12_^−1^ = 8(ω + Δω)^3^β^2^η^3^/εhc^3^. The terms ω and Δω represent the location of the energy-level and bandwidth of pseudo-thermal source, respectively, which can be dressed by the coupling field *G***_1_** and *G***_2_**.

Figure 2(a1,b1) shows the experimental setup for measuring the second- and third-order temporal intensity noise correlation, respectively. We investigated interference in second- and third-order correlation by treating the fourth-order FL as a pseudo-thermal source generated by exciting ***E*_1_** and ***E*_2_** beams on the NV^−^ center. Two independent FL beams pass through non-polarizing beam splitters BS_1_ and BS_2_, and then are detected by three detectors D_1_, D_2_, and D_3_. The output of the detector is input into the three-mode coincidence count system (CCC). We measured the second- and third-order interference of two pseudo-thermal sources. Figure 2(b2) shows that there are eight different cases to emit two modes by two independent sources, which are named as source indistinguishable terms. The first one is all three modes emitted by S_f_. The second one is mode A and B emitted by S_f_, and mode C emitted by S_F_. Other possible source indistinguishable terms are shown in Figure 2(b2). Although the frequencies of the modes emitted by two pseudo-thermal sources are different, these different alternatives can be regarded as indistinguishable if the time measurement uncertainty of the detection system is less than $1/\omega_{ij}$ [21], where $\omega_{ij}$ is the frequency difference of the two sources. In each case, there are six different ways to trigger a three-mode coincidence count (as shown in Figure 2(b3)), which are defined as path indistinguishable terms. The combination of source and path indistinguishable terms results in interference of three modes. The three-mode intensity noise correlation with time delay can be obtained as follows [22]:

(2) $$\begin{array}{l} G^{\left(3\right)}(\tau_{1},\tau_{2},\tau_{3})\propto sinc^{2}\frac{\Delta \omega_{f}\tau_{1}}{2}+sinc^{2}\frac{\Delta \omega_{f}\tau_{2}}{2}+sinc^{2}\frac{\Delta \omega_{f}\tau_{3}}{2}\\ \;+2sinc\frac{\Delta \omega_{f}\tau_{1}}{2}sinc\frac{\Delta \omega_{F}\tau_{1}}{2}*d_{1}+2sinc\frac{\Delta \omega_{f}\tau_{2}}{2}sinc\frac{\Delta \omega_{F}\tau_{2}}{2}*d_{2}\\ \;+2sinc\frac{\Delta \omega_{f}\tau_{3}}{2}sinc\frac{\Delta \omega_{F}\tau_{3}}{2}*d_{3}+2sinc\frac{\Delta \omega_{f}\tau_{1}}{2}sinc\frac{\Delta \omega_{f}\tau_{2}}{2}sinc\frac{\Delta \omega_{F}\tau_{3}}{2}*d_{3}\\ \;+2sinc\frac{\Delta \omega_{f}\tau_{1}}{2}sinc\frac{\Delta \omega_{F}\tau_{2}}{2}sinc\frac{\Delta \omega_{f}\tau_{3}}{2}*d_{2}+2sinc\frac{\Delta \omega_{F}\tau_{1}}{2}sinc\frac{\Delta \omega_{f}\tau_{2}}{2}sinc\frac{\Delta \omega_{f}\tau_{3}}{2}*d_{1} \end{array}$$

In the above equation, sinc(x)=sinx/x and *τ* is the time difference of two photons arrived at detectors. *τ*_1_ = *t*_1_ − *t*_2_, τ_2_ = *t*_2_ − *t*_3_, τ_3_ = *t*_3_ − *t*_1_, and $\Delta \omega_{F/f}=\Delta_{i}^{2}\Gamma_{i}/\Omega^{2}+G_{j}^{2}\Gamma_{i}/\Omega^{2}$ is fluorescence signal’s frequency bandwidth corresponding to its central frequency. In the above equation, $d_{1}=cos\left(t_{1}-t_{2}\right)(\omega_{f}-\omega_{F})$, $d_{2}=cos\left(t_{2}-t_{3}\right)(\omega_{f}-\omega_{F})$, and $d_{3}=cos\left(t_{3}-t_{1}\right)(\omega_{f}-\omega_{F})$ are the frequency beating terms. If we switch off D_3_, the second-order temporal correlation between the detectors D_1_ and D_2_ can be obtained as follows:

(3) $$\begin{array}{l} G^{\left(2\right)}(\tau_{1})\propto \frac{2\Delta \omega_{f}{}^{2}}{r_{0}^{4}}(1+sinc^{2}\frac{\Delta \omega_{f}\left(t_{1}-t_{2}\right)}{2})+\frac{2\Delta \omega_{F}{}^{2}}{r_{0}^{4}}(1+sinc^{2}\frac{\Delta \omega_{F}\left(t_{1}-t_{2}\right)}{2})\\ \;+\frac{4\Delta \omega_{f}\Delta \omega_{F}}{r_{0}^{4}}\left[1-sinc\frac{\Delta \omega_{f}\left(t_{1}-t_{2}\right)}{2}sinc\frac{\Delta \omega_{F}\left(t_{1}-t_{2}\right)}{2}cos(t_{1}-t_{2})(\omega_{f}-\omega_{F})\right] \end{array}$$

From Equations (2) and (3), it can be concluded that interference in intensity–noise correlation depends upon superposition of probable amplitudes of source and path indistinguishable terms.

## 4. Results and Discussion

**Figure 3:**
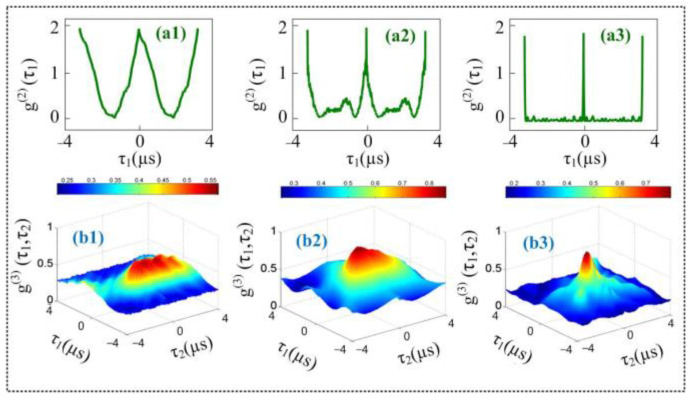
(**a**) Measured second-order temporal correlation of two pseudo-thermal sources plotted by changing the power of ***E*_1_** (*P_E_*_1_) by fixing *t*_1_ time offset at 0 μs; (**a1**) *P_E_*_1_ *=* 1 mW; (**a2**) *P_E_*_1_ *=* 3 mW; (**a3**) *P_E_*_1_ = 5 mW; (**b**) The third-order temporal correlation of two pseudo-thermal sources by changing the power of *E*_1_ (*P_E_*_1_); (**b1**) *P_E_*_1_ = 1 mW; (**b2**) *P_E_*_1_ = 3 mW; (**b3**) *P_E_*_1_ = 5 mW.

Herein, we investigated interference in two- and three-mode intensity noise correlation by treating fourth-order FL from the NV^−^ center as pseudo-thermal sources (S_f_ and S_F_). The fourth-order FL signals are generated by two beams ***E*_1_** and ***E*_2_** in a V-type level system (see Figure 1b). Figure 3a shows interference in the two-mode correlation function $G^{\left(2\right)}(\tau_{1})$(plotted as a function of time delay $\tau_{1}$) when *t*_1_ time offset is fixed at 0 μs and power of ***E*_1_** is changed from low (1 mW) to high (5 mW). In the current experiment, the frequencies of two pseudo-thermal sources are almost degenerate $\omega_{f}-\omega_{F}\approx 0$, and the frequency beating term $cos(t_{i}-t_{j})(\omega_{i}-\omega_{j})$ is approximately equal to 1. Thus, the correlation function mainly shows two-mode bunching peaks, as shown in Figure 3(a1). According to Equation (3), the waveform of the correlation function is determined by ${sinc}^{2}\left[\Delta \omega \left(t_{1}-t_{2}\right)\right]$ and can be controlled by the bandwidth Δω of pseudo-thermal source. When the power of ***E*_1_** is changed from 1 mW to 5 mW, the two dominant peaks, which are assessed by the superposition of the two two-mode bunching, interfere constructively and the waveform of $G^{\left(2\right)}(\tau_{1})$ is changed from broad (Figure 3(a1)) to sharp (Figure 3(a3)). When the power of ***E*_1_** is increased, the splitting space between the dressed energy levels (ω + Δω) increases owing to the strong dressing effect $\left\{{\left|G_{2}\right|}^{2}/\left[\Gamma_{00}+{\left|G_{1}\right|}^{2}/\left(\Gamma_{20}+i\Delta_{1}\right)\right]\right\}$ mentioned in Equation (1). As a result of population redistribution in the dressed state, the lifetime decreases and bandwidths of the corresponding peaks increase, as shown in Figure 3(b3). As the bandwidth Δω of two pseudo-thermal sources is gradually increased, the period of the term ${sinc}^{2}\left[\Delta \omega \left(t_{1}-t_{2}\right)\right]$ in Equation (3) gradually decreases. As a result, the bunching peak becomes more sharp, as shown in Figure 3(a3). Figure 3(b1–b3) shows a similar interference phenomenon for third-order correlation as described for second-order correlation in Figure 3(a1–a3).

The optical demultiplexer from interference between source and path indistinguishable terms is realized by the correlation results observed in Figure 3. Our experiment provides a physical mechanism to control the channel capacity of the optical demultiplexer in delayed time by manipulating the laser power. From our experiment results, the channel capacity can be defined as $C_{t}=(\tau_{+}-\tau_{-})/(\tau_{+}+\tau_{-})$ (where *τ*_+_ and *τ*_−_ are two points at an equal distance on the correlation curve), then $C_{\tau }=89\%$ when ***E*_1_** power is set at 1 mW and its deturning is set at resonance. This can be explained by the higher lifetime and bandwidth, which results in a broad lineshape of the correlation function.

**Figure 4:**
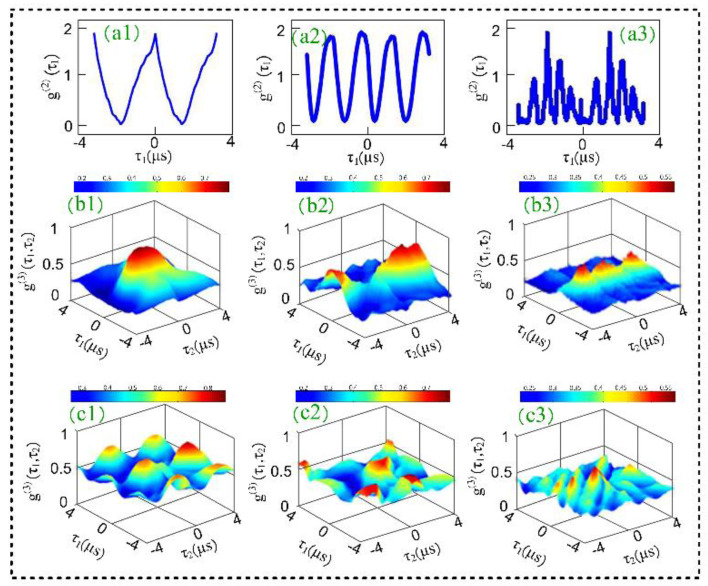
(**a**) The second-order temporal correlation of two pseudo-thermal sources by changing *t*_1_ time offset when the power of ***E*_1_** is fixed at 1 mW. (**a1**) *t*_1_ = 0 μs; (**a2**) *t*_1_ = 1 μs; (**a3**) *t*_1_ = 2 μs; (**b**,**c**) The third-order temporal correlation of two pseudo-thermal sources when when the power of ***E*_1_** is fixed at 1 mW and 3 mW, respetively. (**b1**) *t*_1_ = 0 μs; (**b2**) *t*_1_ = 1 μs; (**b3**) *t*_1_ = 2 μs; (**c1**) *t*_1_ = 0 μs; (**c2**) *t*_1_ = 1 μs; (**c3**) *t*_1_ = 2 μs.

Figure 4 shows second- and third-order temporal correlation functions at different *t*_1_ time offset (0, 1, and 2 μs) by fixing the power of ***E*_1_** at 1 mW. The remaining experimental conditions (the laser’s detuning and Rabi frequency) are the same as reported in Figure 3. When *t*_1_ time offset is fixed at 0 μs (Figure 4(a1)), the correlation curve shows a broadened peak, which is caused by quantum interference between two- and three-mode bunching, as in the discussion concerning Figure 2. When *t*_1_, *t*_2_, and *t*_3_ time offset are equal (0 μs each), the three-mode bunching amplitudes interfere constructively, and the third-order correlation function $G^{\left(3\right)}(\tau_{1},\tau_{2},\tau_{3})$ mentioned in Equation (2) achieves its maximum value. When *t*_1_ time offset is increased to 2 μs, the single dominant peak is converted into three secondary peaks owing to the increase in quantum interference among the three types of two-mode bunching and one type of three-mode bunching, as shown in Figure 4(a3). From the above discussion, it can be concluded that three-mode bunching dominates at *t*_1_ time offset at 0 μs and three-two mode bunching becomes dominant at *t*_1_ time offset at 2 μs.

The demultiplexer from the interference of two- and three-mode bunching is realized by the correlation results observed in Figure 4. Our experiment provides a physical mechanism to realize the optical 1 ∗ 4 demultiplexer (shown in Figure 1c) in the delayed time domain by manipulating time offset. In Figure 4(b1–b3), the “bright (peak)” and “dark (no peak)” modes correspond to logic 1 and logic 0, respectively. In this device, ‘S_0_′ and ‘S_1_′ are two selection lines and each correlation curve in Figure 4 corresponds to unique output (O_1_–O_4_) of the demultiplexer. By controlling two selection lines S_0_ (*t*_1_ time offset) and S_1_ (*t*_2_ time offset), the output of the demultiplexer can be controlled. When S_0_ = S_1_ = 0 (both *t*_1_ and *t*_2_ time offset are zero), then the output of the demultiplexer would be “0100” as a single bright state is observed (Figure 4(b1)). When S_0_ = 1 and S_1_ = 0, then the measured output of the demultiplexer would be “1001”, as two dark states are sandwiched between two bright states (Figure 4(b2)). The operational performances of this logic device turned out to be the same as the truth table for the demultiplexer proposed in Figure 1d. Channel spacing is the difference between adjacent channels in any system. The greater the value of channel spacing, the greater the accuracy of information and the less interference between channels. Channel spacing for correlation curves can be defined as $\eta =(\tau^{\prime }-\tau )/(\tau^{\prime }+\tau )$, then η=96% (Figure 4(b3)) for *t*_1_ time offset 2 μs. The total time delay between switching from one output to another output is measured at 17 ns, and is taken as the quadrature sum of several independent contributions.

**Figure 5:**
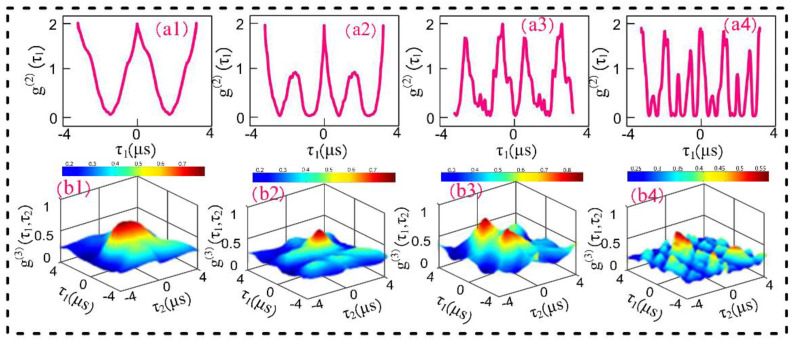
(**a**) The second-order temporal correlation of two pseudo-thermal sources; (**a1**) S_f_ wavelenth = 575 nm; (**a2**) S_f_ wavelenth = 576 nm; (**a3**) S_f_ wavelenth = 577 nm; (**a4**) S_f_ wavelenth = 578 nm; (**b**) The third-order temporal correlation of two pseudo-thermal sources, the experimental conditons of (**b1**–**b4**) are same as that of (**a1**–**a4**), respectively.

In Figure 5, correlation curves are plotted by varying the wavelength of pseudo-thermal source S_f_ from 575 nm to 637 nm, and fixing S_F_ at 575 nm. When two pseudo-thermal sources are almost degenerate (Figure 5(b1)), i.e., $\omega_{i}\approx \omega_{j}$, three-mode bunching is dominant, and the frequency beating term $cos(t_{i}-t_{j})(\omega_{i}-\omega_{j})$ is approximately equal to 1. Thus, the third-order correlation function mainly shows a dominant center peak caused by three-mode bunching. As the frequency of pseudo-thermal source S_f_ increases, the interference term $cos(t_{i}-t_{j})(\omega_{i}-\omega_{j})$ becomes more prominent in the temporal correlation function in Equation (3). We can see that the dominant center peak becomes sharper and, simultaneously, the secondary peaks are enhanced as shown in Figure 5(b3). The secondary peaks can be attributed to quantum path interference between the three two-mode mode bunching and one three-mode bunching. With the frequency of source S_f_ increasing further, the correlation peak shows strong interference peaks and the number of peaks increases dramatically, as illustrated in Figure 5(b4). This result is caused by the combined effect of three-mode bunching and second- and third-order quantum beating. From Equations (2) and (3), we can see that interferences in both second- and third-order correlation are determined by $cos(t_{i}-t_{j})(\omega_{i}-\omega_{j})$. The oscillation frequency of secondary peaks is proportional to the frequency deference $\left(\omega_{i}-\omega_{j}\right)$. Thus, the number of peaks increases with the increase in frequency deference.

The interferometer model is realized by the correlation results observed in Figure 5. The interference index of the interferometer ($H=I_{\mathrm{int}}/I_{bun}$) is the ratio of amplitude of the next lower peak ($I_{int}$) to the amplitude of bunching peak ($I_{bun}$). The interference index provides us with an accurate insight into the quantum path interference between the two-mode bunching and three-mode bunching. In our experiment, the interference index H increases from 0.01 (Figure 5(b1)) to 0.9 (Figure 5(b3)) as the wavelength of the pseudo-thermal source S_f_ changes from 575 nm to 637 nm.

## 5. Conclusions

In conclusion, we have explored the correlation function temporally between two pseudo-thermal sources in the NV^−^ center. Based on the temporal correlation function of the two pseudo-thermal sources, we demonstrate a physical model of an optical demultiplexer. The channel spacing and speed of the demultiplexer can reach about 96% and 17 ns, respectively. It will have potential applications in quantum computing and communication.
